# Cell factory for γ-aminobutyric acid (GABA) production using *Bifidobacterium adolescentis*

**DOI:** 10.1186/s12934-021-01729-6

**Published:** 2022-03-07

**Authors:** Hend Altaib, Tomoya Kozakai, Yassien Badr, Hazuki Nakao, Mahmoud A. M. El-Nouby, Emiko Yanase, Izumi Nomura, Tohru Suzuki

**Affiliations:** 1 Laboratory of Genome Microbiology, Faculty of Applied Biological Sciences, Gifu University, 1-1 Yanagido, Gifu, 501-1193 Japan; 2 The United Graduate School of Agricultural Science, Gifu University, 1-1 Yanagido, Gifu, 501- 1193 Japan; 3 Department of Animal Medicine, Faculty of Veterinary Medicine, Damanhour University, El-Beheira, Egypt; 4 Department of Pesticide Chemistry and Technology, Faculty of Agriculture, Alexandria University, El-Shatby, Alexandria, 21545 Egypt; 5 Graduate School of Engineering, Gifu University, 1-1 Yanagido, Gifu, 501-1193 Japan

**Keywords:** *Bifidobacterium adolescetis*, GABA, Fed batch fermentation

## Abstract

**Background:**

Bifidobacteria are gram-positive, probiotic, and generally regarded as safe bacteria. Techniques such as transformation, gene knockout, and heterologous gene expression have been established for *Bifidobacterium*, indicating that this bacterium can be used as a cell factory platform. However, there are limited previous reports in this field, likely because of factors such as the highly anaerobic nature of this bacterium. *Bifidobacterium adolescentis* is among the most oxygen-sensitive *Bifidobacterium* species. It shows strain-specific gamma-aminobutyric acid (GABA) production. GABA is a potent bioactive compound with numerous physiological and psychological functions. In this study, we investigated whether *B. adolesentis* could be used for mass production of GABA.

**Results:**

The *B. adolescentis* 4–2 strain isolated from a healthy adult human produced approximately 14 mM GABA. It carried *gadB* and *gadC*, which encode glutamate decarboxylase and glutamate GABA antiporter, respectively. We constructed pKKT427::P_*ori*_*-gadBC* and pKKT427::P_*gap*_*-gadBC* plasmids carrying *gadBC* driven by the original *gadB* (*ori*) and *gap* promoters, respectively. Recombinants of *Bifidobacterium* were then constructed. Two recombinants with high production abilities, monitored by two different promoters, were investigated. GABA production was improved by adjusting the fermentation parameters, including the substrate concentration, initial culture pH, and co-factor supplementation, using response surface methodology. The optimum initial cultivation pH varied when the promoter region was changed. The *ori* promoter was induced under acidic conditions (pH 5.2:4.4), whereas the constitutive *gap* promoter showed enhanced GABA production at pH 6.0. Fed-batch fermentation was used to validate the optimum fermentation parameters, in which approximately 415 mM GABA was produced. The conversion ratio of glutamate to GABA was 92–100%.

**Conclusion:**

We report high GABA production in recombinant *B. adolescentis*. This study provides a foundation for using *Bifidobacterium* as a cell factory platform for industrial production of GABA.

**Supplementary Information:**

The online version contains supplementary material available at 10.1186/s12934-021-01729-6.

## Introduction

Microbial cell factories are bioengineered cells used for cost-efficient production of recombinant proteins and valuable chemicals, such as amino acids and vitamins [1, 2]. *Escherichia coli* has been used as an efficient transformation platform for many years; however, detoxifying the produced recombinant proteins remains difficult. *Escherichia coli* is a gram-negative bacterium that contains lipopolysaccharides (LPS), which is known as an endotoxin, in its outer membrane. Lipopolysaccharide induces a pyrogenic response and ultimately triggers septic shock in mammalian cells [3, 4]. Therefore, it is necessary to eradicate lipopolysaccharide before applying the products in medicine or food; however, the eradication process can be costly [5]. Therefore, new, safe, and efficient production platforms have been investigated [6]. Use of Gram-positive bacterial species simplifies the purification of recombinant proteins [7]. Lactic acid bacteria (LAB) have also been extensively studied for this purpose [7, 8].

Bifidobacteria are symbiotic bacteria that are widely used as probiotics in health-promoting foods and supplements. They are promising alternative platforms for chemical production because of their safety and gram-positive and endotoxin-free nature which allow for easy purification, and the current availability of gene manipulation tools. Furthermore, Ninomiya and colleagues reported that a 100% CO_2_ supply is sufficient to promote growth and material production by bifidobacteria and assists in maintaining the anaerobic condition of the production culture [9]. Together, this culture can be scaled up for eco-friendly industrial production, eliminating the costs related to the sterilized air supply and vigorous agitation.

Gamma-aminobutyric acid (GABA) is a four-carbon amino acid synthesized from glutamate by pyridoxal-5′-phosphate (PLP)-dependent glutamate decarboxylase (GAD; EC 4.1.1.15) [10] and acts as a major inhibitory neurotransmitter in the central nervous system [11]. Moreover, GABA shows potential as a supplement for treating many conditions, including depression, poor sleep quality [12, 13], poor immunity [14], and diabetes, as it strongly promotes insulin secretion [15, 16]. Thus, various approaches for producing GABA have gained attention in recent years [8]. Biotransformation based on microbial biosynthesis is an eco-friendly approach for producing valuable biomolecules, such as GABA, compared to chemical biosynthesis.

Metabolic engineering can be used to develop microbes with novel phenotypes that are optimized to function as microbial cell factories [17]. Several gene manipulation tools have become available for *Bifidobacterium* species [18]. We previously reported the construction of a *Bifidobacterium*-*E. coli* shuttle vector pKKT427, a plasmid artificial modification method (PAM) [19], and the development of a gene knockout technique using temperature-sensitive plasmids [20]. We also recently conducted an analytical study of the core promoters of *Bifidobacterium* [21]. These tools have provided insight into *Bifidobacterium *spp*.* The current study was conducted to use available genetic tools to produce an industrially relevant phenotype of *Bifidobacterium*, focusing on GABA as a model biochemical.

## Results

### GABA production by wild-type *B. adolescentis*

*Bifidobacterium adolescentis* 4–2 is a fecal strain of *Bifidobacterium* isolated from healthy adult humans living in Japan [22]. The whole genome of this strain was analyzed (Accession number: BPPZ01000001–BPPZ01000033), and annotation analysis revealed that in wild-type *B. adolescentis* 4–2, GABA is produced through the functions of two genes, *gadB* (Accession number: LC598719) and *gadC* (Accession number: LC598720), encoding glutamate decarboxylase and GABA-glutamate antiporter, respectively. The wild-type *B. adolescentis* 4–2 strain produced approximately 14 mM GABA after 72 h of fermentation in De Man, Rogosa, Sharpe (MRS) medium containing 67 mM monosodium glutamate (MSG), with an average production rate of 0.02 g/L/h (Additional file 1: Fig. S1).

### GABA production by recombinant *Bifidobacterium* strains

**Fig. 1:**
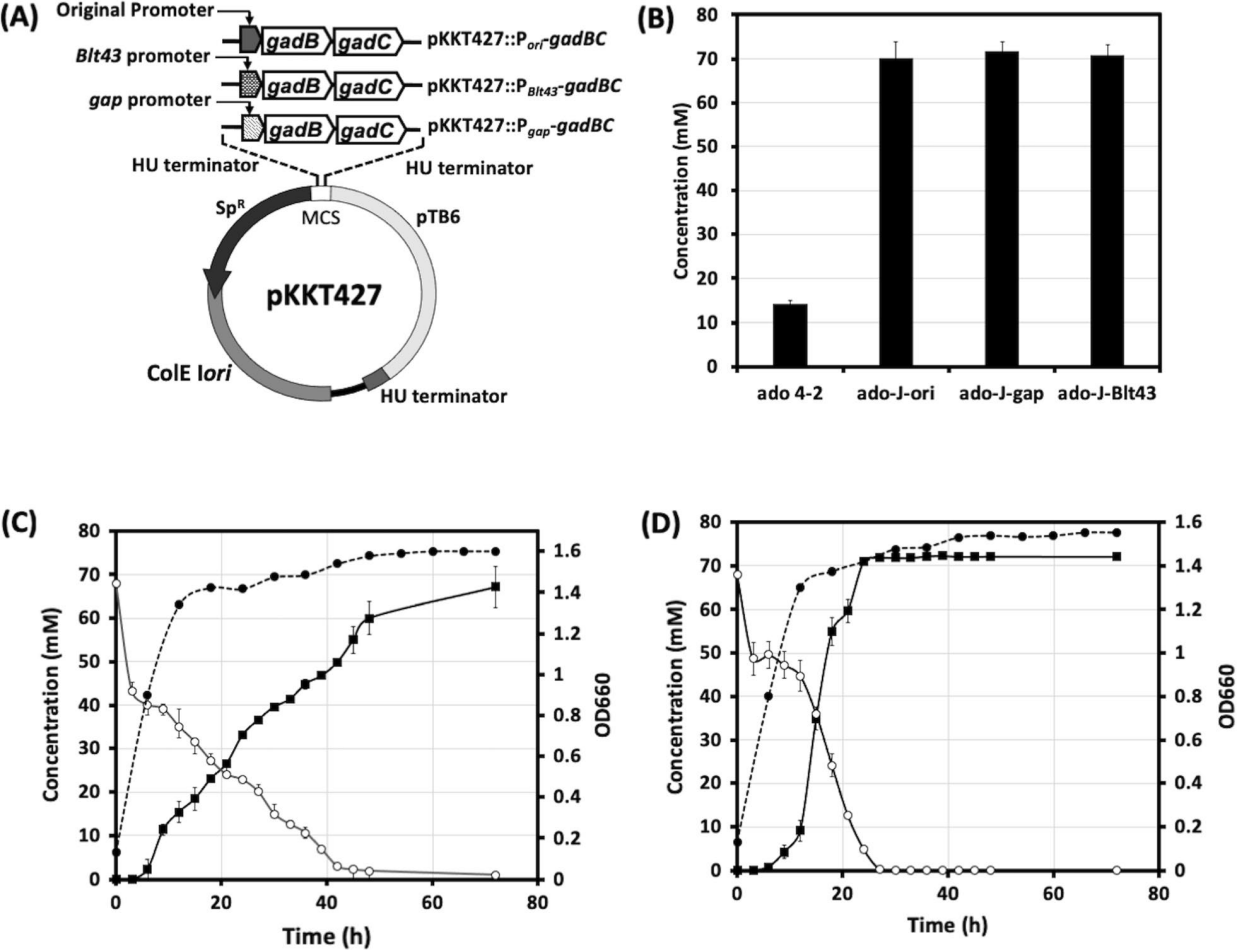
Cloning plasmid and GABA production by each *Bifidobacterium* recombinant. **A** Diagram of expression vector construction displaying the backbone of pKKT427, a *Bifidobacterium*-*E*. *coli* shuttle vector, in which GABA-producing genes were inserted within the multiple cloning site (MCS). The names of three plasmid constructs with different promoters are mentioned in the upper part of the MCS. **B** Glutamate/GABA conversion by *B. adolescentis* strains; *B. adolescentis* 4–2 wild (ado.4–2), *B. adolescentis* JCM 1275/pKKT427::P_*Ori*_*-gadBC* (ado-J-ori), *B. adolescentis* JCM 1275/pKKT427::P_*Ori*_*-gadBC* (ado-J-gap), *B. adolescentis* JCM 1275/pKKT427::P_*Blt43*_*-gadBC* (ado-J-Blt43). **C**, **D** Bacterial growth and glutamate/GABA conversion pattern in two recombinant strains. **C** *B. adolescentis* JCM 1275/pKKT427::P_*Ori*_*-gadBC*. **D** *B. adolescentis* JCM 1275/pKKT427::P_*gap*_*-gadBC*. Bacterial growth (optical density, OD600; ●), GABA production (mM; ■), and glutamate concentration (mM; ○) are displayed. Values are presented as the means ± SD. Analysis was performed using three independent bacterial cultures

GABA-producing genes were overexpressed in non-GABA-producing hosts (Additional file 2: Table S1). Three promoters, including two constitutive promoters (*gap*, the glyceraldehyde 3-phosphate dehydrogenase gene, and *Blt43*, a tRNA gene) and the original *gadB* promoter (*ori*), were tested for driving the expression of *gadBC* (Fig. 1A). The *gap* and *Blt43* promoters have been reported to efficiently enhance gene expression in *Bifidobacterium* [23, 24]. The conversion ratio of glutamate to GABA in recombinant *B. adolescentis* JCM 1275 exceeded 100% when the cells were grown in MRS medium containing 67 mM MSG (Fig. 1B), which was sufficient for further analysis. The conversion ratio was calculated using the following equation:

$$ \text{Conversion ratio} =\frac{final\,GABA \,production\left(mM\right)}{Initial \,glutamate\, concentration\left(mM\right)}\times 100$$

To validate the efficiency of GABA production in other non-GABA-producing *Bifidobacterium* species, *gadBC* overexpression vectors were cloned into three non-GABA-producing hosts of *Bifidobacterium*, *B. longum* 105-A, *B. longum* subsp. *infantis* JCM 1222, and *B. minimum* JCM 5821, each of which has different characteristic features [25, 26] (Table 1). GABA was detected in all recombinants and produced at different levels depending on the promoter efficiency. The original promoter showed low efficiency in strains other than *B. adolescentis*, suggesting that strain-specific factors affected the original promoter activity. The other two constitutive promoters more efficiently expressed *gad*. The conversion ratio of glutamate to GABA ranged from 84 to 97%. GABA production ranged from 55 to 67 mM when the cells were grown in MRS containing 67 mM MSG among high GABA-producing *Bifidobacterium* recombinants (Additional file 2: Table S1, Additional file 3: Fig. S2).

**Table 1:** *Bifidobacterium* strains and promoters used in this study

	Strain/promoter/plasmid	Characteristic feature/origin	References
Bacterial Strains	*B. adolescentis* 4-2	Wild type GABA producer, isolated from adult human feces living in Japan	[22]
	*B. adolescentis* JCM 1275	Low transformation efficiency, high oxygen sensitivity, isolated from adult human feces, obtained from JCM company	[19]
	*B. longum* 105-A	High transformation efficiency, isolated from adult human gut	[25]
	*B.* *longum* subsp. *infantis* JCM 1222	High oxygen sensitivity, isolated from infant gut	[26]
	*B. minimum* JCM 5821	Unique oxygen tolerance, isolated from sewage	[26]
	*E. coli* TOP10	Chemically competent cells	
Promoters	P_*gap*_	The promoter of Glyceraldehydes-3-phosphate dehydrogenase gene, it is a part of *B. longum* 105-A genome	[23]
	P_*BLt43*_	The promoter of tRNA gene, it is a part of *B. longum* NCC2705 genome	[24]
	P_*ori*_	The promoter of glutamate decarboxylase gene, it is a part of *B. adolescentis* 4-2 genome	This study
Plasmids	pKKT427	A shuttle vector between *Escherachia coli* and *Bifidobacterium* with Sp^r^, modified of pBRATA101	[19]
	pBCMAT_P_*gap*__T_*dppA2*_	A plasmid construct for Chloramphenicol assay based on pKKT427 backbone, including *gap*-promoter	[24]
	pBCMAT_P_*BLt43*__T_*dppA2*_	A plasmid construct for Chloramphenicol assay based on pKKT427 backbone, including *Blt43*-promoter	[24]
	pKKT427::P_*ori*__*gadBC*	pKKT427 carrying *gadB* and *gadC* genes with the original promoter *gadB* gene	This study
	pKKT427::P_*gap*__*gadBC*	pKKT427 carrying *gadB* and *gadC* genes with the *gap*-promoter	This study
	pKKT427::P_*BLt43*__*gadBC*	pKKT427 carrying *gadB* and *gadC* genes with *Blt43*-promoter	This study
	pPAM1233-1283	pBAD33 carrying BAD_1233 and BAD_1283 PAM plasmid, used for methylation of pKKT427	[19]

Because of their high glutamate/GABA conversion ratio, *B. adolescentis* JCM 1275/pKKT427::P_*ori*_*-gadBC* (R1) and *B. adolescentis* JCM 1275/pKKT427::P_*gap*_*-gadBC* (R2) were further investigated. In R1, GABA was continuously produced at 48–72 h of fermentation (Fig. 1C), whereas in R2, GABA was produced within 18–24 h (Fig. 1D). Furthermore, during the exponential phase, the GABA production rate in R2 was higher than that in R1 (0.2 vs. 0.1 g/L/h, respectively).

The stability of GABA production by the two recombinant strains was investigated. Both R1 and R2 displayed similar activity for GABA production after one year of storage, confirming their stability during long-term storage (Additional file 4: Fig. S3).

### Optimum conditions for GABA production from *B. adolescentis* JCM 1275/pKKT427::P_*ori*_-*gadBC*

**Fig. 2:**
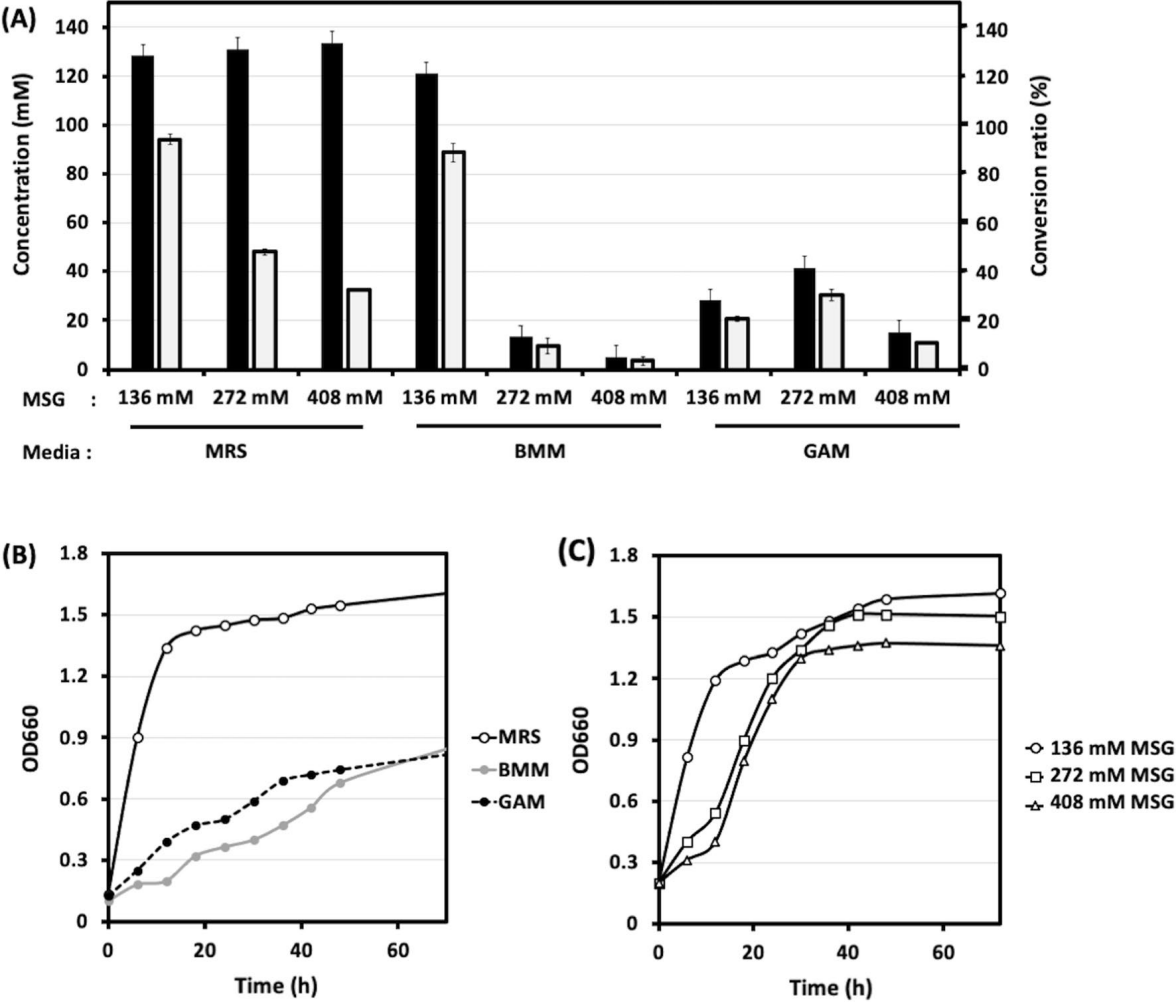
GABA production and bacterial growth on different media. **A** Effect of media on glutamate/GABA conversion in *B*. *adolescentis* JCM 1275/pKKT427::P_*ori*_*-gadBC*. Three media and different concentrations of monosodium glutamate (MSG) (%, v/v) were tested. Media names and MSG % are displayed at the bottom of the graph. **B** Bacterial growth on De Man, Rogosa, Sharpe (MRS), Bifidobacterial minimal medium (BMM), and Gifu Anaerobic Medium (GAM) media. **C** Bacterial growth in MRS medium with different concentrations of MSG. Values are presented as the means ± SD. Analysis was performed using three independent bacterial cultures

The effects of the media, substrate concentration, and pH on GABA production were investigated. Both MRS and Bifidobacterial minimal medium (BMM) [27] showed comparable GABA productivities at 136 mM MSG. MSG concentrations higher than 136 mM caused a slight reduction on bacterial growth and did not increase GABA production in MRS medium (pH 6.5 ± 0.3) (Fig. 2). In BMM, higher MSG concentrations decreased both bacterial growth and GABA production (Fig. 2A, B), likely because of the change in osmolarity of the medium [28]. Gifu anaerobic medium (GAM), a medium developed for general culture and susceptibility testing of anaerobic bacteria, showed the lowest concentrations of GABA (Fig. 2A). Hence, the MRS medium showed the best result for both bacterial growth and GABA production (Fig. 2), it was used in all subsequent analyses for both R1 and R2.

**Fig. 3:**
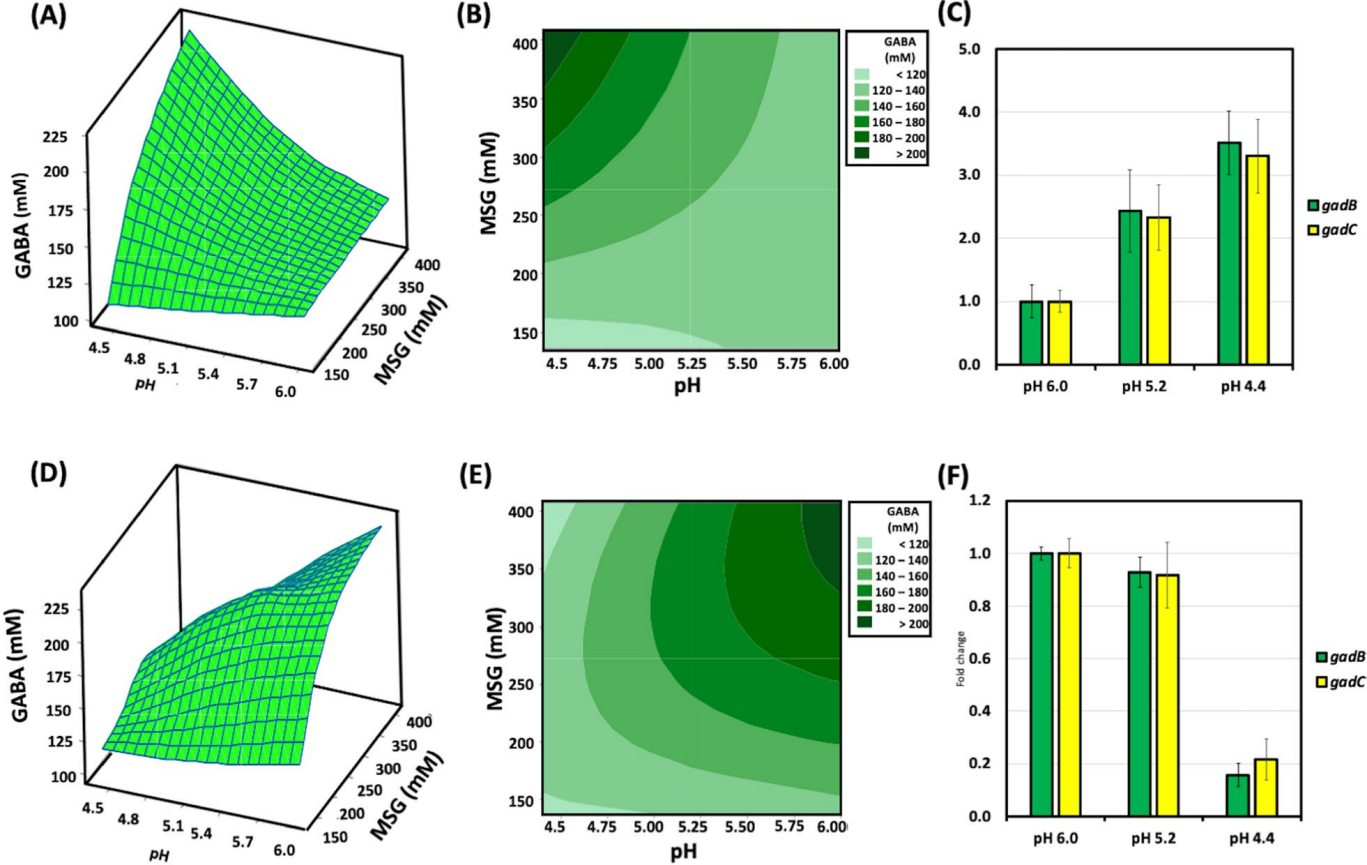
Response surface plot (**A**, **D**) and contour plot (**B**, **E**) to examine the effect of substrate concentration and initial culture pH on GABA yield. **A**–**C** *B. adolescentis* JCM 1275/pKKT427::P_*Ori*_-*gadBC.* **D**–**F** *B. adolescentis* JCM 1275/pKKT427::P_*gap*_-*gadBC.* **C**, **F** *gadB* and *gadC* expression at different initial culture pH levels. Values are presented as the means ± SD

To estimate the effect of the culture pH and MSG concentration on extracellular GABA production, response surface methodology (RSM) was conducted to match the two factors. A pH range of 4.0–6.0 and an MSG concentration of 100 to 400 mM were evaluated. Figure 3A and B show the surface and contour plots for the mixed effects of both the pH and MSG concentration. The results suggest that acidic pH (4–5) improves extracellular GABA production in this recombinant.

### Optimum conditions for GABA production from *B. adolescentis* JCM1275/pKKT427::P_*gap*_***-****gadBC*

A multi-factorial design was used to test the effect of both the culture pH and MSG concentration on extracellular GABA production. Interestingly, the results suggested that the optimal pH for extracellular GABA production was nearly neutral (pH 6.0) (Fig. 3D, E), possibly because of changes in the promoter activity from *ori* to *gap*. To validate this finding, *gadB* and *gadC* expression was evaluated at different pH values. In R1, both *gadB* and *gadC* showed higher expression at acidic pH (4.4 and 5.2) than at pH 6.0 (Fig. 3C). In contrast, R2 showed higher expression at pH 6.0 than at acidic pH (4.4 and 5.2) (Fig. 3F).

### PLP enhances GABA production

As PLP is an essential co-factor for GABA production, it may recover *gad* activity, particularly at later stages of bacterial growth. Hence, we investigated the effect of PLP addition on extracellular GABA at the optimum initial culture pH for R1 and R2. PLP was added at 0, 24, and 48 h during bacterial growth. For R1, when PLP was added at 24 and 48 h of fermentation, GABA production was much higher than when PLP was added at 0 h (Additional file 5: Fig. S4A). In R2, when PLP was added at 24 h of fermentation, GABA production was much higher than when PLP was added at 0 h. However, addition of PLP after 48 h did not result in a significant increase in GABA production compared to that at 0 h (Additional file 5: Fig. S4 B). These results suggest that PLP addition partially recovered *gad* activity, which was also detectable by RSM for GABA production after adding PLP to the fermentation medium (Additional file 6: Fig. S5).

### Fed-batch fermentation model for enhanced GABA production from both recombinants R1 and R2

**Fig. 4:**
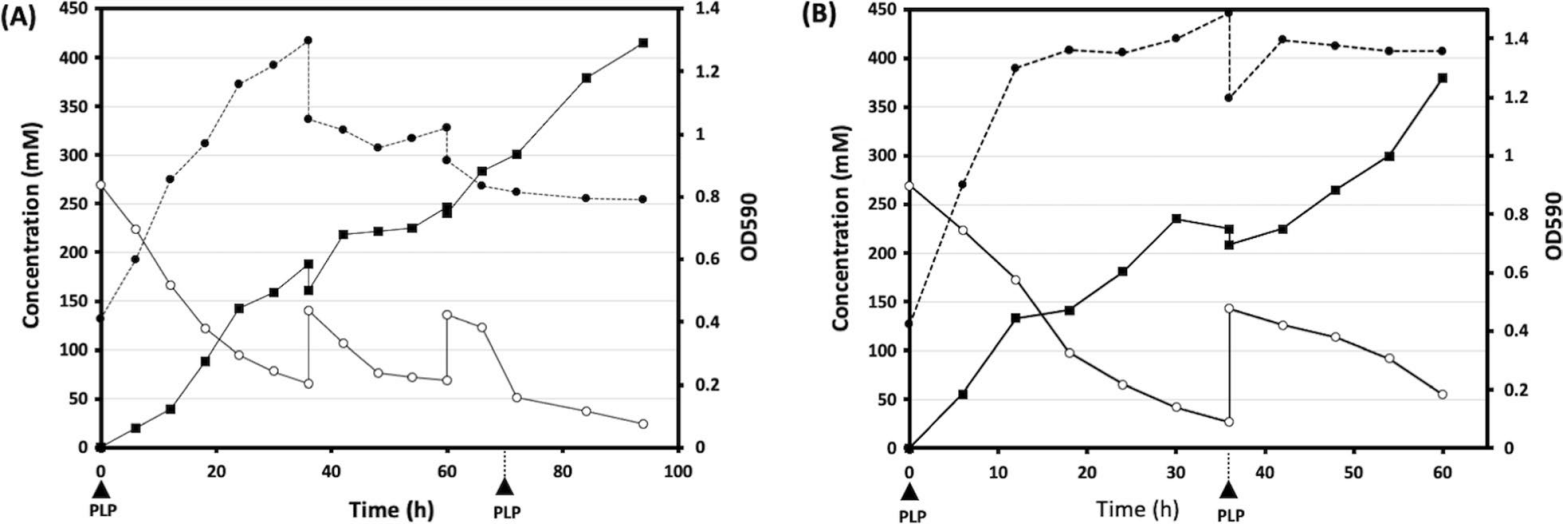
Evolution of GABA production (mM; ■), glutamate concentration (mM; ○), and biomass production (OD580; ●) during growth of two recombinant strains of *Bifidobacterium*. **A** *B. adolescentis* JCM 1275/pKKT427::P*ori*-*gadBC* in De Man, Rogosa, Sharpe (MRS) containing an initial concentration of 270 mM of monosodium glutamate (MSG) at initial pH of 4.4. Approximately 70 mM MSG was added twice at 39 and 60 h of fermentation. Pyridoxal 5′-phosphate (PLP) (0.05 µM) was added twice at 0 and 72 h. **B** *B. adolescentis* JCM 1275/pKKT427::P*gap*-*gadBC* in MRS containing an initial concentration of 270 mM of MSG at initial pH of 6.0. Approximately 110 mM MSG was added once at 39 and 60 h of fermentation. PLP (0.05 µM) was added twice at 0 and 36 h

Fed-batch fermentation was performed for *B. adolescentis* JCM 1275/pKKT427::P*ori*-*gadBC* grown in MRS medium containing 270 mM MSG (Fig. 4A). The initial pH of the culture was adjusted to 4.4. When the glutamate level was reduced to 67 mM, additional glutamate was added and dissolved in the MRS medium at pH 4.4. PLP (0.5 µM) addition times were determined based on the GABA concentration in the medium, which was continuously monitored. The total added MSG, estimated as 415 mM, was fully converted to GABA in this fermentation model, producing 415 mM GABA after 98 h of incubation.

Another model was used for *B. adolescentis* JCM 1275/pKKT427::P*gap*-*gadBC* grown in MRS medium containing 270 mM MSG (Fig. 4B). The culture pH was maintained at approximately 6.0 during the first 12 h to ensure a high cell number; the pH was not monitored. When the glutamate level was reduced to approximately 30 mM, additional glutamate was added to the MRS medium at pH 6.0. PLP (0.5 µM) was added at 0 and 36 h based on the GABA concentration. The total added MSG was approximately 390 mM, almost all of which was converted to GABA in this fermentation model, leading to the production of approximately 380 mM GABA after only 60 h of incubation.

## Discussion

We constructed GABA-producing recombinants of *Bifidobacterium* and developed an efficient fermentation scheme to scale-up GABA production in *B. adolescentis*, with GABA production reaching (415 mM) 42 g/L. These results suggest that this bacterium is an efficient cell factory platform.

*Bifidobacterium adolescentis* JCM 1275 possesses a type II restriction-modification system that acts as a barrier against foreign DNA, such as plasmid DNA. This system limits the use of *B. adolescentis* JCM 1275 as an expression host [29]; however, the PAM system overcomes this limitation, as the transformation efficiency was improved to 10^4^ colony-forming units and plasmid stability was increased [19]. Furthermore, *B. adolescentis* was recently reported as an advantageous candidate for gene expression and recombination [24]. In our study, recombinants of *B. adolescentis* JCM 1275 achieved a high GABA production efficiency exceeding a 100% conversion ratio of glutamate to GABA. This likely reflects the ability of these recombinants to produce small amounts of glutamate, which is converted to GABA via glutamate decarboxylase activity. Furthermore, both R1 and R2 showed high production stability even after one year of storage, which is advantageous for repeated utilization regardless of the storage time. In our study, recombinants of *B. adolescentis* JCM 1275 outperformed the wild type *B. adolescentis* 4-2 in terms of GABA productivity, which is likely due to increased gene copy number via overexpression and improved plasmid stability by using PAM system. The Original *gadBC* promoter does not improve GABA productivity in recombinants of *B. longum* 105 A, *B. infantis* JCM 1222 and *B. minimum* JCM 5821 compared to *B. adolescentis* JCM 1275. These data suggests that the original *gadBC* promoter of *B. adolescentis* most likely requires unknown trans-acting activator.

The fermentation parameters were optimized for the two recombinants, R1 and R2. These recombinants showed similar high GABA productivity but in different fermentation conditions (Fig. 3). The observed difference may be attributed to differences in each promoter used to drive *gadBC.* The *gap* promoter in R2 led to strong and constitutive expression of *gadBC* but was suppressed at acidic pH (Fig. 3F). In contrast, the *gadBC* promoter in R1 induced *gadBC* expression at acidic pH (Fig. 3C). These effects were reflected in the GABA productivity, as shown in RSM analysis. To estimate gene expression levels in both R1 and R2, 16SrRNA was used as an internal standard to normalize *gadB* and *gadC* expression. Although 16SrRNA is a multi-copy gene, it has been used as an internal standard in several previous investigations [30–32]. The expression of internal standard genes may differ based on the experimental conditions [33]. In our study, 16SrRNA showed equal expression under different pH conditions (Additional file 7: Fig. S6). Therefore, 16SrRNA was used as an internal standard in our experiments.

Numerous factors affect the ability of lactic acid bacteria and bifidobacteria to produce GABA [34]. Maintaining a high bacterial density may increase the yield of GABA through the production of glutamate decarboxylase [35]. Figure 2 shows that the MRS medium was optimal for both bacterial growth and GABA production compared to BMM and GAM. MRS medium is more nutrient-rich than BMM and GAM media. For instance, the glucose content in MRS medium (2%) was much higher than that in GAM medium (0.3%). Although BMM is a synthetic medium that is suitable for production because of its low cost and ease of purification, further improvements are needed to enhance productivity using this medium.

Glutamate decarboxylase catalyzes the irreversible decarboxylation of glutamate to GABA, which is activated by the coenzyme PLP. Therefore, adding a small amount of PLP to the culture medium can restore the activity of the GAD system. In our study, addition of PLP effectively restored GABA production. Yang and colleagues observed a similar effect in a subspecies of *Streptococcus salivarius*, with PLP addition during the stationary phase leading to increased GABA production because of the easy denaturation of PLP in the early growth phases [28]. In contrast, addition of PLP to the fermentation media of gut-derived *B. adolescentis* 150 did not affect GABA production in a previous study [34]. This difference may be related to the ability of some bacteria to generate PLP through decarboxylation of pyridoxal aldehyde; however, the bacteria-produced PLP cannot meet the requirement for recombinant production.

RSM has been used successfully in several studies to determine the optimum conditions for GABA yield in *Lactobacillus* species [36, 37]. In our study, the optimum conditions for achieving a high GABA yield in R1 and R2 were similar for the concentrations of MSG (415 mM) and PLP (0.5 µM), whereas the optimum pH differed for the two tested recombinants (R1, pH 4.4; R2, pH 6.0). R2 produced more GABA at neutral pH, which is also optimal for bacterial growth.

Fed-batch fermentation is an efficient approach to validate the optimum fermentation parameters [38]. Two-step batch fermentation has been reported for several lactobacilli [39, 40]. In our study, R1 and R2 efficiently converted glutamate to GABA following a single pH adjustment throughout the cultivation period. Bifidobacteria multiplication is affected by oxygen and hydrogen peroxide, and cell viability decreases at pH values below 4.0 [41–43]. Therefore, our strategy for promoting the high viability of bifidobacteria in fed-batch fermentation at acidic pH involved using a large number of viable cells during initial fermentation and performing all processes under anaerobic conditions. In addition, GABA and glutamate levels were monitored during fermentation, and the timing of PLP addition was determined based on the GABA concentration. This system led to the accumulation of GABA in a yield of 42 g/L (415 mM).

Previous reports have described numerous efficient cell factories for GABA production, including *E. coli*, which is among the commonly used hosts in laboratory-scale metabolic engineering. Enzymatic production of GABA in *E. coli* can reach 103 g/L/h [44]. However, in some cases, proteins must be purified from *E. coli* to eradicate toxic LPS contamination [5]. Therefore, studies have been performed to broaden the number of microbial cell factories [6]. Lactic acid bacteria effectively produce GABA. For instance, *Lactobacillus lactis* NCDO2118 produced up to 413 mM GABA from 441 mM glutamate [45]. The GABA production ability varied among bacterial species and strains in the range of 2.9–614 g/L (Additional file 2: Table S2) [44–58]. The recombinant *B. adolescentis* produced in our study produced an intermediate level of GABA compared to other reported GABA producers. Broad adjustment of fermentation parameters may enhance GABA productivity to reach the level of high producers.

Many studies have focused on GABA production from lactic acid bacteria because of its value in both industrial production [8] and food processing [59–61]. These studies showed that various lactic acid bacteria have different fermentation requirements and different GABA production abilities. Human-derived bifidobacteria were previously screened for their ability to produce GABA under different conditions [34]; however, a combination of all tested parameters has not been validated. In our study, three key factors, MSG, PLP, and pH, were optimized using RSM, and the combination of optimum fermentation parameters was validated using batch fermentation. The optimal conditions established in this study for *B. adolescentis* recombinants can be used for further development of functional GABA-containing food and microbial cell factories using *Bifidobacterium*.

*Bifidobacterium* has advantages and disadvantages as a cell factory platform. For instance, the usage of this gram-positive bacterium eliminates the cost of protein purification, which is required when using gram-negative bacteria such as *E. coli*. Additionally, Bifidobacteria are anaerobic bacteria, eliminating the cost of vigorous agitators required for aerobic production; thus, use of this bacterium can lower production costs. Further, *Bifidobacterium* does not contain endotoxins or inclusion bodies that affect the fermentation product. Furthermore, techniques required for the recombination and engineering of bifidobacteria have become available [18]. Gene expression in different *Bifidobacterium* species has also been investigated [21, 24]. One limitation of using *Bifidobacterium* is the extreme oxygen sensitivity of some species; however, in this study, we used the extremely oxygen-sensitive species *B. adolescentis* and successfully obtained stable and high GABA production. In this study, we overexpressed a gene encoded by a simple cluster, composed of two genes. Further studies are needed to express proteins or biochemicals encoded by complex pathways in *Bifidobacterium*. We focused on basic fermentation parameters affecting GABA productivity. Other parameters, such as osmolarity, should be further examined.

## Conclusions

Previously, researchers regarded *Bifidobacterium* as a host that is difficult to use in industrial production. Particularly, use of *B. adolescentis* is impractical in this field because of its high oxygen sensitivity. We applied available genetic tools and techniques to enable the use of *B. adolescentis* as an efficient cell factory platform.

We improved GABA production from recombinant *B. adolescentis* to approximately 42 g/L. Our findings provide valuable insights for the broad application of GABA-producing *Bifidobacterium* in various fields, particularly on the industrial scale.

## Methods

### Bacterial strains, plasmids, and cultivation conditions

The bacterial strains, plasmids, and promoters used in this study are listed in Table 1. Luria-Bertani (LB) medium was used to cultivate *E. coli* TOP10 competent cells (Life Technologies, Carlsbad, CA, USA). MRS medium (Difco, Detroit, MI, USA) was used for standard cultivation of *Bifidobacterium*. Other media used were GAM (Nissui Pharmaceutical, Tokyo, Japan), developed for general culture and susceptibility testing of anaerobic bacteria and BMM as a chemically defined medium containing inorganic salts, glucose, vitamins, isoleucine, and tyrosine.

*Bifidobacterium* was manipulated under anaerobic conditions on a BUG Box (Ruskinn Technology, Bridgend, UK) using a mixed gas supplement (80% N_2_, 10% CO_2_, and 10% H_2_). MSG (Sigma Aldrich, Louis, MO, USA) was added to the liquid bacterial culture as a substrate for GABA production. *Bifidobacterium adolescentis* 4-2 was isolated from the feces of healthy adults. Bacterial isolation and identification were previously described [22]. Informed written consent was obtained from all study participants. The study was approved by the Institutional Ethics Committee of Gifu University.

### Tube cultivation

Standard cultivation was performed by inoculating the frozen stock (−80 °C) into liquid preculture, followed by incubation at 37 °C for 24 h. The bacteria were then sub-cultured in MRS medium at different initial pH values with different concentrations of MSG. After 48 or 72 h of incubation, the bacteria were centrifuged at 6000 rpm for 3 min. The supernatant was used for high-pressure liquid chromatography (HPLC) analysis. Spectinomycin (Sp) (75 µg/mL) was added to both the *Bifidobacterium* and *E. coli* recombinant cultures.

### Fed-batch fermentation

Small-scale batch cultivation (100 mL) was used to simulate the tube culture in a large volume of medium. Ten milliliters of the precultured cells were centrifuged and sub-cultured in 100 mL MRS medium containing 270 mM MSG, 75 µg/mL Sp, and 0.5 mM PLP. When the glutamate concentration was less than 70 mM, fresh MRS medium containing additional MSG was added to the culture. PLP (0.5 mM) was added when the GABA concentration did not show a relative increase during batch fermentation. One milliliter of the sample was collected every 6 h, from which 300 µL was used for absorbance measurement, 100 µL was used for HPLC analysis, and the remaining 600 µL was used for pH assessment. The pH of the culture was adjusted every 12 h as necessary.

### Optimization of fermentation parameters for GABA production

The optimal combination of the media type, MSG concentration, initial pH, incubation time, and bacterial growth was determined by measuring the amount of extracellular GABA produced under each condition. MRS, BMM, and GAM were tested as culture media, to which different concentrations of MSG were added, and GABA production was estimated. The effect of the initial pH was assessed by adjusting the pH to values to 4.4–6.0 using HCl or NaOH. Bacterial growth and GABA production were monitored for each parameter.

### Response surface methodology

A complete factorial design was used in RSM to estimate the optimum conditions for GABA production. Two independent factors (f), glutamate concentration, and pH were investigated at three levels (n): low, high, and medium, which were coded as −1, +1, and 0, respectively (Additional file 2: Table S3). The obtained results were fitted to a quadratic polynomial regression model using linear, quadratic, and two-factor interactions for *B. adolescentis* JCM 1275:: P*ori*-*gadBC* (1) and *B. adolescentis* JCM 1275::P*gap*-*gadBC* (2).

1 $$ {\text{Y}}\, = \,{{184}}\, + \,{{1}}.{{814}} X_{i}  - 90.8 X_{{ii}}  - 0.{{2281}} X_{i} \, \times \,X_{{ii}}  - 0.000{{799}}X_{i} ^{{{2}}} \, + \,{{12}}.{{25}} X_{{ii}} ^{{{2}}} $$

2 $$ {\text{Y}}\, = \, - {{162}} - 0.00{{9}}X_{i} \, + \,{{74}}.{{8}}X_{{ii}} \, + \,0.{{2468}} X_{i} \, \times \,X_{{ii}}  - 0.00{{2}}0{{35}}X_{i} ^{{{2}}}  - {{8}}.{{68}} X_{{ii}} ^{{{2}}} $$

Y is the response GABA (mM) and Xi and Xii are the two variables of glutamate concentration (mM) and pH, respectively. The various assemblies used are listed in Additional file 2: Table S4. The results of analysis of variance using the regression equation are shown in Additional file 2: Tables S5 and S6 for R1 and R2, respectively.

### Molecular cloning and DNA manipulation

The oligonucleotide primers are listed in Table 2. Genomic DNA was extracted from *B. adolescentis* 4-2 using an Isofaecal DNA extraction kit (Nippon Gene, Toyama, Japan). Plasmids were extracted from *E. coli* using a QIAprep Spin Mini Kit (QIAGEN, Hilden, Germany). *gadB* and *gadC* were amplified using KOD-Plus-Neo polymerase (Toyobo, Osaka, Japan). Golden Gate cloning, a type IIS enzyme-based strategy [62, 63], was used for plasmid construction. The Type IIS restriction enzyme *Sap*I was used. The recognition site of the *Sap*I restriction enzyme was added to both the forward and reverse primers used to amplify the *gadBC* and pKKT427 backbone (Table 2). The thermal cycle program for Golden Gate cloning was performed as previously described [64]. The plasmid constructs were transformed into *E. coli* cells and selected on LB (Sp) agar. The transformed plasmids were then extracted and verified by sequencing using the BigDye Terminator ver. 3.1 Cycle and ABI 3130xl (Applied Biosystems, Foster City, CA, USA). The correct plasmid construct was transformed into *Bifidobacterium* using electroporation (MicroPulser, Bio-Rad, Hercules, CA, USA) as previously described [20]. Transformants were selected on MRS agar containing Sp (75 µg/mL). The pKKT427 plasmid was methylated before transformation into *B. adolescentis* JCM 1275 using the PAM method as previously described [19]. Briefly, pKKT427::P_*gap*_*-gadBC-*sp and PAM plasmid (pBAD33-cm containing methylation genes) were co-transformed into *E. coli* TOP10 cells. A single colony was subcultured in LB broth containing L-arabinose (1%), Sp (75 µg/mL), and chloramphenicol (15 µg/mL) to allow for methylation. The plasmid was then extracted and electroporated into *B. adolescentis* JCM 1275.

**Table 2:** Oligonucleotides used in the study

Primer	Sequence (5’—> 3’)	Template	Purpose
gadBC_OP_Fw	ccagctcttcgACAacctgcccatcgtagc	*B. adolescentis* 4–2 genomic DNA	Amplify *gadBC* gene with the original promoter
gadBC_OP_RV	ccagctcttcgCTAtcagtattccggattcactagc		
pKKT427 Fw	caagctcttcgTAGgccaccgtcgccaagg	pKKT427	Amplify pKKT427 plasmid backbone
pKKT427 Rv	caagctcttcgTGTgcctgcatgcaagctt		
gadBC Fw	ccagctcttcgatgtcagaaacacattccacc	*B. adolescentis* 4–2 genomic DNA	Amplify *gadBC* gene without the original promoter
gadBC Rv	caagctcttcgtcagtattccggattcactagc		
pKKT427_ter_Fw	ccagctcttcgTGActgactcactgaacgg	pBCMAT_P*gap*_TdppA2	Amplify pKKT427 plasmid backbone, including the *gap* promoter and terminator
pKKT427_pro_rv	ccagctcttcgCATgatgttctccttgggtca		
gadB_RT1_Fw	catgttcctgcgtttgggat	*B. adolescentis* cDNA	*gadB* quantitative expression
gadB_RT1_Rv	ccgtcgttccacagcgta		
gadC_RT1_Fw	cgtcggtttcgtcgctt		*gadC* quantitative expression
gadC_RT1_Rv	cacaagaatcgcatatgaaacgcta		
16srRNA_FW	cacattccaccgttacacc		Normalize gene expression in *B. adolescentis*
16srRNA_FW	cgttatccggaattattggg		

Underlined sequences are *SapI* recognition site

### Real-time PCR and mRNA manipulation

Total RNA was extracted from *Bifidobacterium* using a TRIzol (Thermo Fisher Scientific, Waltham, MA, USA)-based method as previously described [21]. Reverse transcription was performed using an iScript^TM^ cDNA Synthesis Kit (Bio-Rad). The resulting cDNA was assessed using real-time PCR on an ABI StepOnePlus system (Applied Biosystems) with the ΔΔCt method [65]. Real-time PCR was performed using the THUNDERBIRD^TM^ SYBR® qPCR mix (Toyobo). The 16S rRNA gene of *B. adolescentis* was used as an internal standard for normalization of expression. The primers used for 16S rRNA, *gadB*, and *gadC* amplification are listed in Table 2. Primers were designed using Oligo ver. 7 software.

### HPLC analysis

The concentrations of GABA and glutamate were determined by HPLC using pre-column fluorescent derivatization with the *o*-phthalaldehyde method as previously described [66]. Briefly, the supernatant of the bacterial culture was filtered through a 0.45-µm micropore filter, diluted by 1000-fold, and 100 µL was mixed with derivatizing solutions and incubated at room temperature for 2 min. The derivatization reaction mix was prepared as described previously [67]. The derivatization product was directly injected into the HPLC system (Agilent Technologies, Santa Clara, CA, USA) with a fluorescence detector (excitation, 350 nm; emission, 450 nm) and Cosmosil packed column 5C_18_-MS-II (3.0ID × 150 mm, Nacalai Tesque, Kyoto, Japan). To prepare the standard curve, GABA (Wako, Osaka, Japan) and glutamate (Sigma) were used. The mobile phase and gradient conditions were maintained as described previously [67].

### Statistical analysis

Experiments were performed in triplicate, and data are expressed as the means ± standard deviation. The actual response value was used to express the GABA yield. The reported data represent the mean values. Surface and contour plots were created using MINITAB® software (v17.1.0, 2002).

## Supplementary Information

**Additional file 1: Figure S1.** GABA production in wild-type *B. adolescentis* 4–2. GABA/glutamate conversation pattern and bacterial growth over 72 h. Bacterial growth (OD600; ●), GABA production (mM; ○), and glutamate concentration (mM; ■) are displayed. Values are presented as the means ± SD.

**Additional file 2: Table S1.** GABA production and glutamate/GABA conversion ratio by different recombinants of *Bifidobacterium.* **Table S2.** Summary of GABA production ability by various microorganisms. **Table S3.** Independent variables and their coded and actual values were used in RSM optimization. **Table S4.** Three-level two-factor full factorial design arrangements and responses. **Table S5.** Analysis of variance (ANOVA) for regression analysis of *B. adolescentis* JCM 1275**/**pKKT427::P_*ori*_-*gadBC.* **Table S6.** Analysis of variance (ANOVA) for regression analysis of *B. adolescentis* JCM 1275**/**pKKT427::P_*gap*_-*gadBC.*

**Additional file 3: Figure S2.** Glutamate/GABA conversion by *Bifidobacterium* recombinant strains, *B. longum* 105-A (lon.), *B. longum subspecies infantis* JCM 1222 (inf.), and *B. minimum* JCM 5821 (min.), each cloned with three promoters. The promoter names are displayed under the corresponding strains. Glutamate and GABA concentrations are presented in g/L. Values are presented as the means ± SD. Analysis was performed using three independent bacterial cultures.

**Additional file 4: Figure S3.** Stability of GABA production from GABA-producing recombinants of *Bifidobacterium adolescentis*. GABA production before and after one year storage of *B. adolescentis* JCM 1275/pKKT427::P_*Ori*_-*gadBC* (A) and *B. adolescentis* JCM 1275/pKKT427::P_*gap*_-*gadBC* (B).

**Additional file 5: Figure S4.** Effect of pyridoxal 5′-phosphate (PLP) addition time on GABA production. (A) *B. adolescentis* JCM 1275/pKKT427::P_*Ori*_-*gadBC* at initial pH of 4.4. (B) *B. adolescentis* JCM 1275/pKKT427::P_*gap*_-*gadBC* at initial pH of 6.0. GABA (mM) is displayed as a solid black line, and glutamate/GABA conversion ratio is shown as a black separated line. Values are presented as the means ± SD. Analysis was performed using three independent bacterial cultures.

**Additional file 6: Figure S5.** Response surface and contour plots depicting γ-aminobutyric acid (GABA) yield, with addition of pyridoxal 5′-phosphate (PLP) by *B. adolescentis* recombinants, *B. adolescentis* JCM 1275/pKKT427::P_*Ori*_-*gadBC* (A, B) and *B. adolescentis* JCM 1275/pKKT427::P_*gap*_-*gadBC* (D, E). The interaction between the initial culture pH and substrate concentration of monosodium glutamate (MSG) (mM) following addition of the co-factor PLP is shown.

**Additional file 7: Figure S6.** The obtained Ct values for 16SrRNA of *B. adolescentis* JCM 1275 recombinant, under different pH conditions.

## Data Availability

The datasets used and analyzed during the current study are available from the corresponding author upon reasonable request.

## Abbreviations

GABA: Gamma amino butyric acid
MRS: De Man Rogosa and Sharpe medium
BMM: Bifidobacterial minimal medium
GAM: Gifu anaerobic medium
LB: Luria-Bertani medium
MSG: Monosodium glutamate
PLP: Pyridoxal 5′-phosphate
Sp: Spectinomycin
HPLC: High-performance liquid chromatography
qPCR: Quantitative PCR
RSM: Response surface methodology
*gadB*: Glutamate decarboxylase
*gadC*: Glutamate/GABA antiporter
R1: *B. adolescentis* JCM 1275:P*ori*-*gadBC*
R2: *B. adolescentis* JCM 1275:P*gap*-*gadBC*
