# Supplementary figures and images for: Cell factory for γ-aminobutyric acid (GABA) production using Bifidobacterium adolescentis
Source: Microb Cell Fact. 2022 Mar 7;21:33. doi: 10.1186/s12934-021-01729-6 (PMC8903651; doi:10.1186/s12934-021-01729-6)

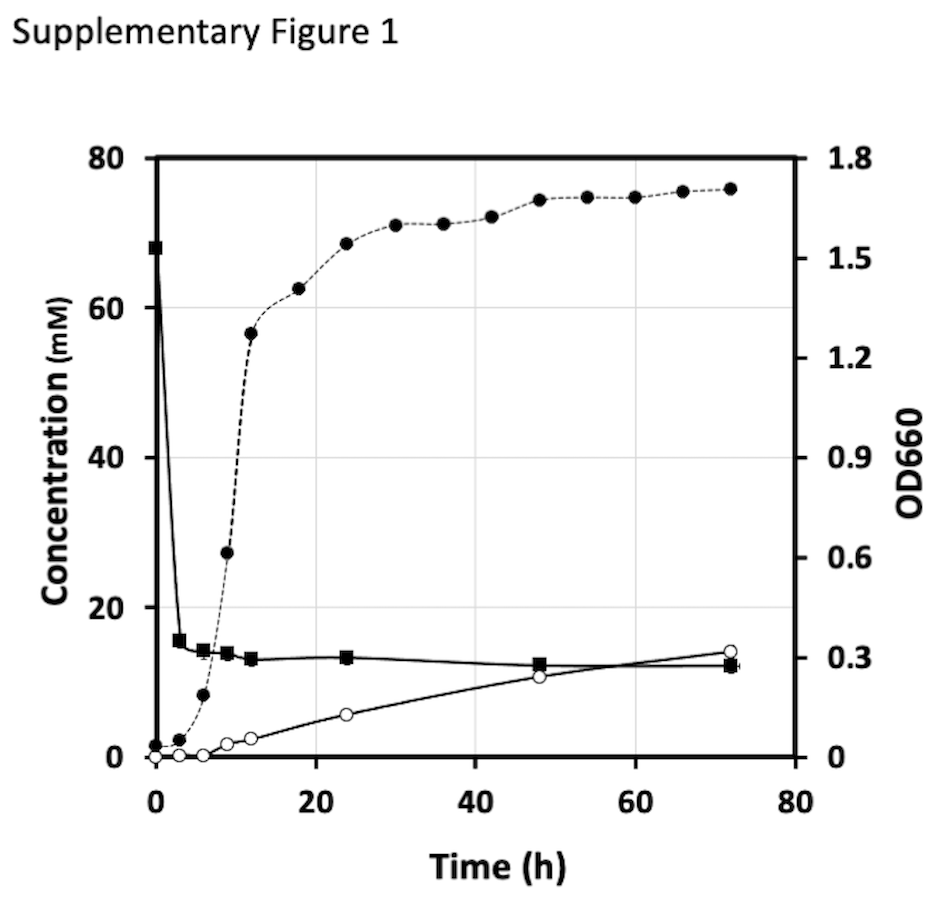

Supplement: Supplementary file 1 — Additional file 1: Figure S1. GABA production in wild-type B. adolescentis 4–2. GABA/glutamate conversation pattern and bacterial growth over 72 h. Bacterial growth (OD600; ●), GABA production (mM; ○), and glutamate concentration (mM; ■) are displayed. Values are presented as the means ± SD. [file 12934_2021_1729_MOESM1_ESM.tiff]

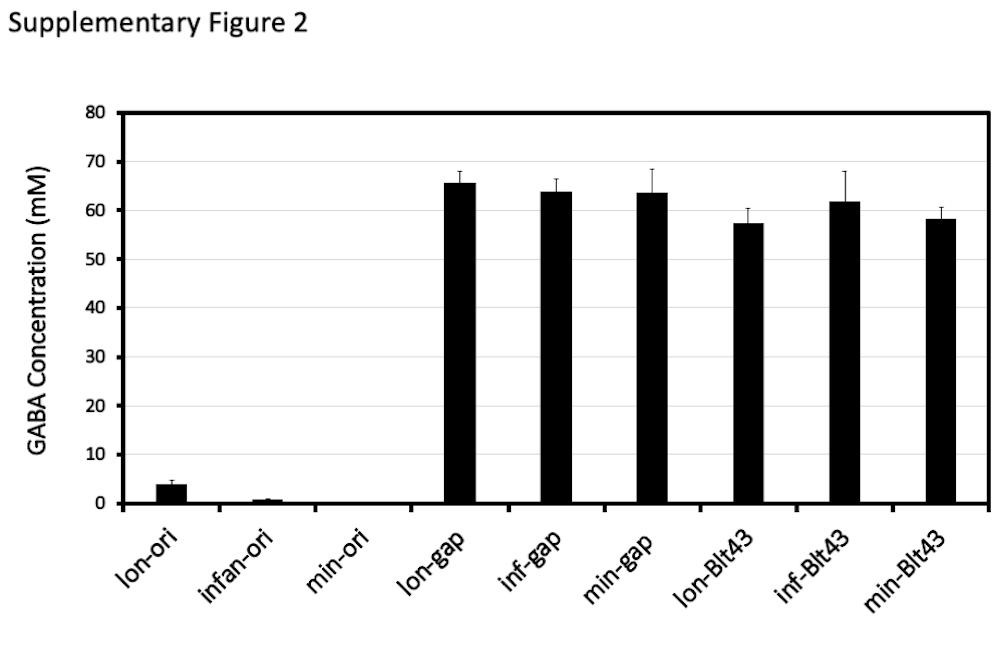

Supplement: Supplementary file 3 — Additional file 3: Figure S2. Glutamate/GABA conversion by Bifidobacterium recombinant strains, B. longum 105-A (lon.), B. longum subspecies infantis JCM 1222 (inf.), and B. minimum JCM 5821 (min.), each cloned with three promoters. The promoter names are displayed under the corresponding strains. Glutamate and GABA concentrations are presented in g/L. Values are presented as the means ± SD. Analysis was performed using three independent bacterial cultures. [file 12934_2021_1729_MOESM3_ESM.tiff]

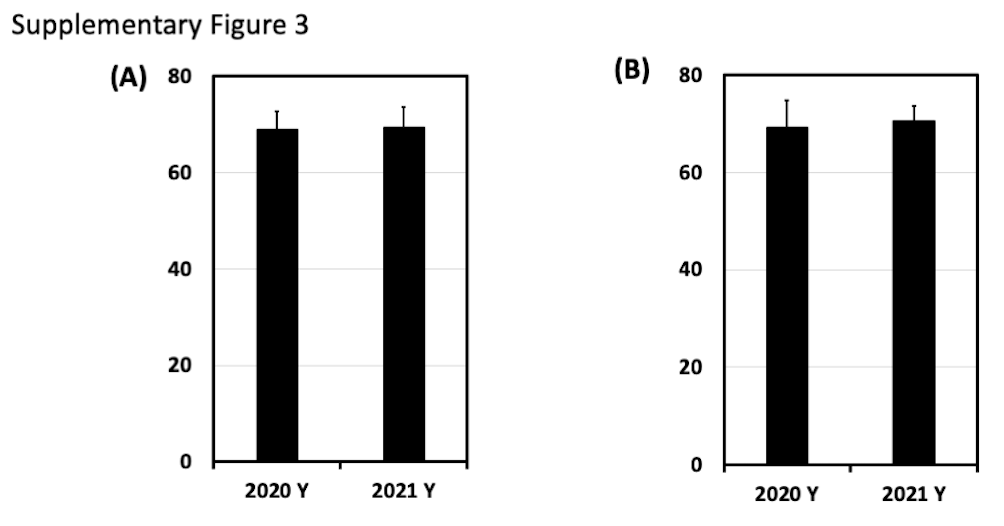

Supplement: Supplementary file 4 — Additional file 4: Figure S3. Stability of GABA production from GABA-producing recombinants of Bifidobacterium adolescentis. GABA production before and after one year storage of B. adolescentis JCM 1275/pKKT427::POri-gadBC (A) and B. adolescentis JCM 1275/pKKT427::Pgap-gadBC (B). [file 12934_2021_1729_MOESM4_ESM.tiff]

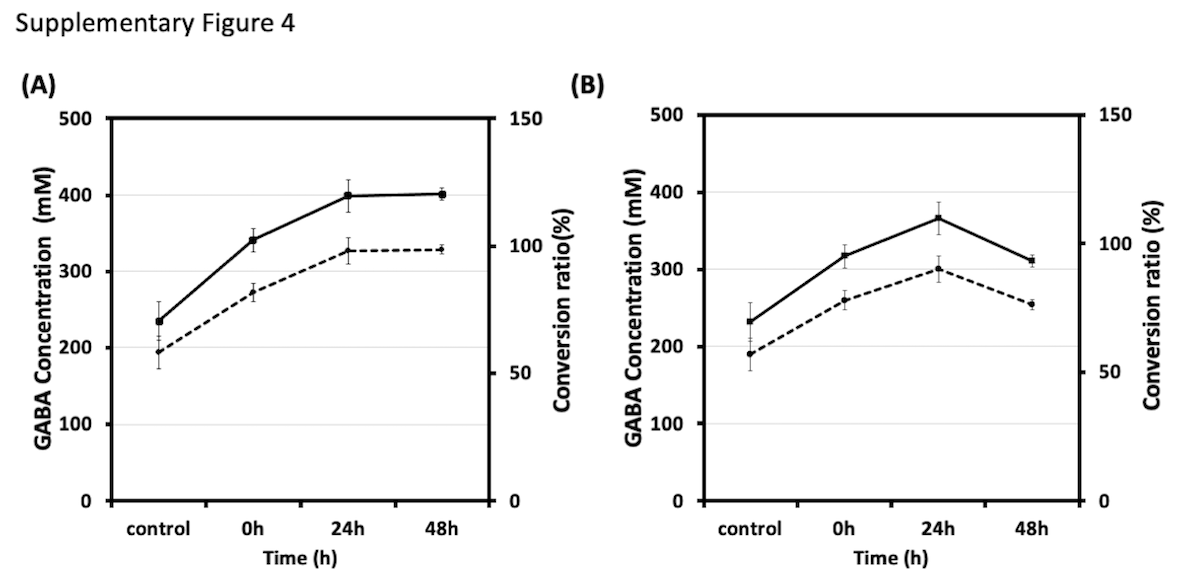

Supplement: Supplementary file 5 — Additional file 5: Figure S4. Effect of pyridoxal 5′-phosphate (PLP) addition time on GABA production. (A) B. adolescentis JCM 1275/pKKT427::POri-gadBC at initial pH of 4.4. (B) B. adolescentis JCM 1275/pKKT427::Pgap-gadBC at initial pH of 6.0. GABA (mM) is displayed as a solid black line, and glutamate/GABA conversion ratio is shown as a black separated line. Values are presented as the means ± SD. Analysis was performed using three independent bacterial cultures. [file 12934_2021_1729_MOESM5_ESM.tiff]

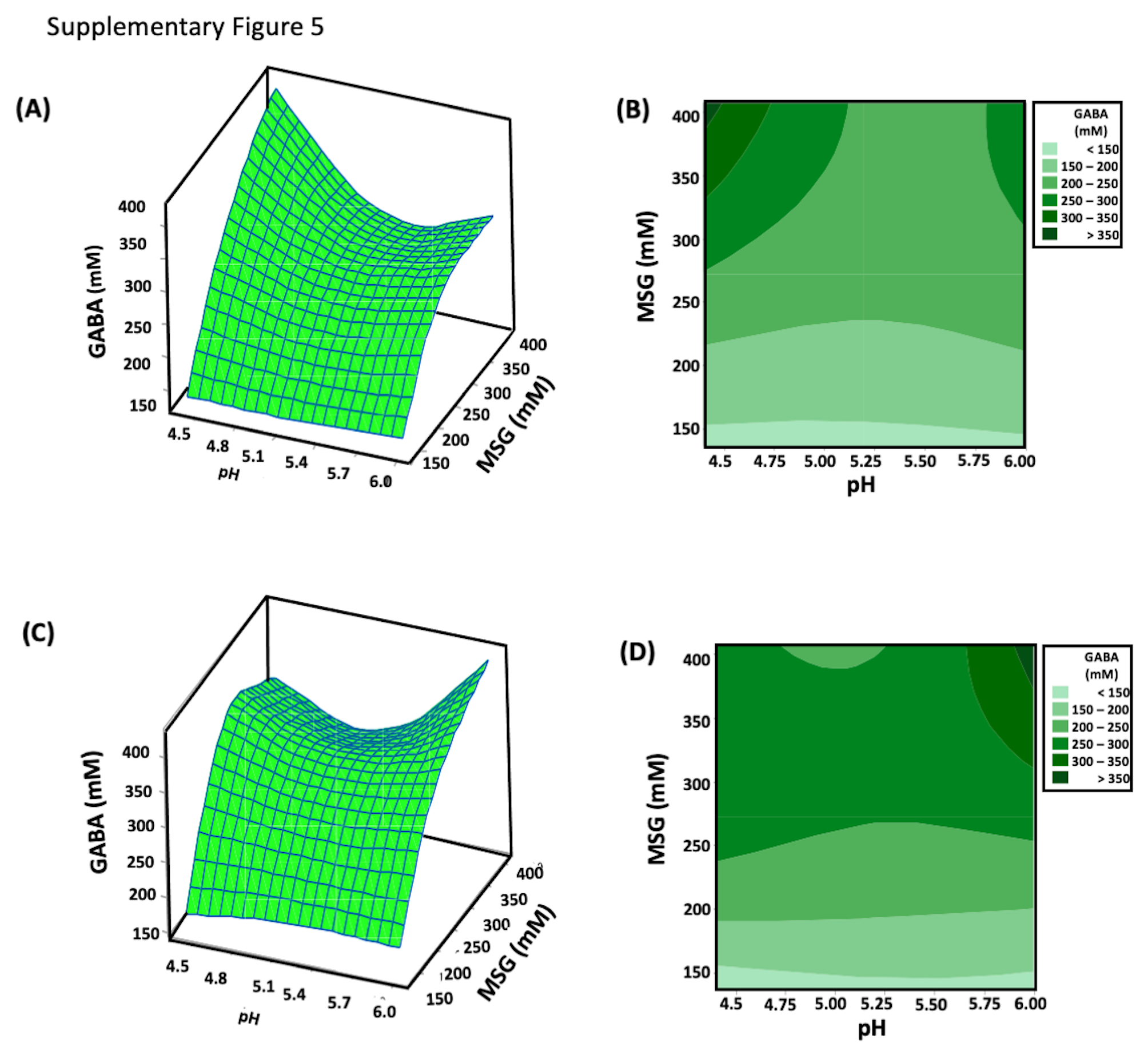

Supplement: Supplementary file 6 — Additional file 6: Figure S5. Response surface and contour plots depicting γ-aminobutyric acid (GABA) yield, with addition of pyridoxal 5′-phosphate (PLP) by B. adolescentis recombinants, B. adolescentis JCM 1275/pKKT427::POri-gadBC (A, B) and B. adolescentis JCM 1275/pKKT427::Pgap-gadBC (D, E). The interaction between the initial culture pH and substrate concentration of monosodium glutamate (MSG) (mM) following addition of the co-factor PLP is shown. [file 12934_2021_1729_MOESM6_ESM.tiff]

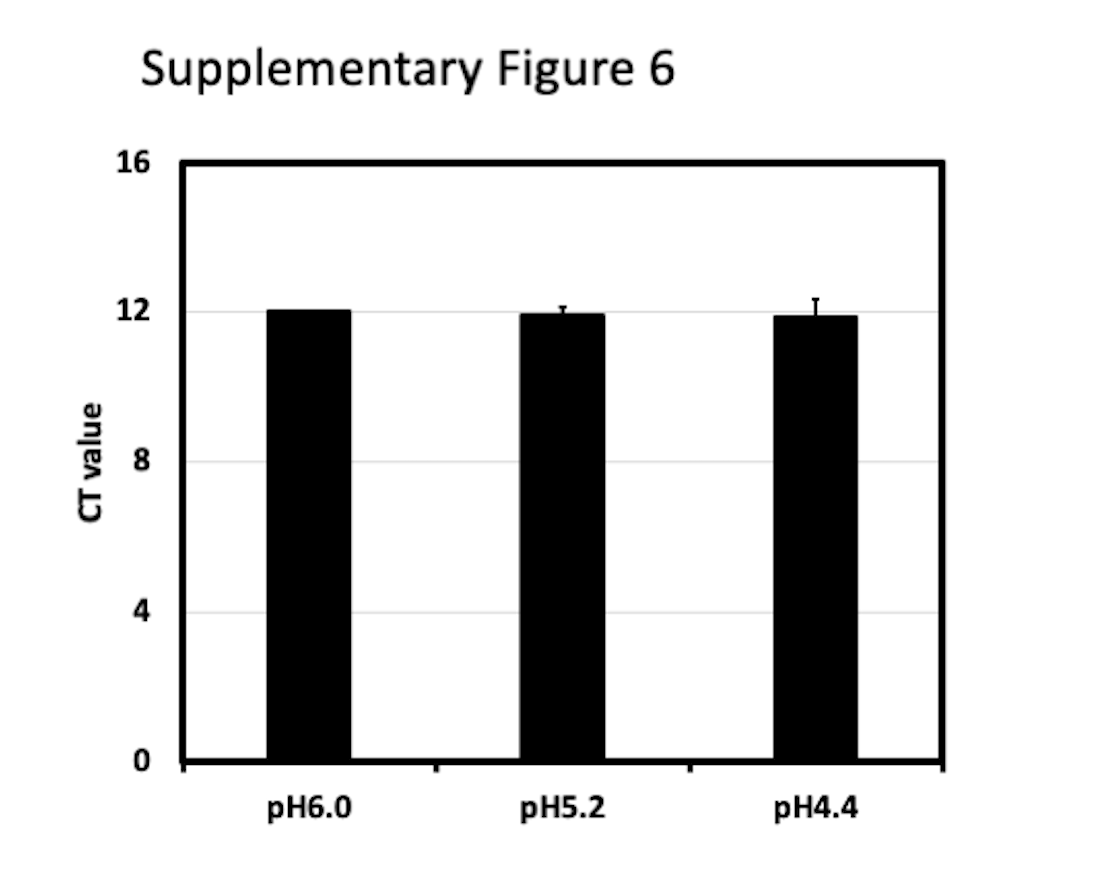

Supplement: Supplementary file 7 — Additional file 7: Figure S6. The obtained Ct values for 16SrRNA of B. adolescentis JCM 1275 recombinant, under different pH conditions. [file 12934_2021_1729_MOESM7_ESM.tiff]
