# Supplementary material for: Cell factory for γ-aminobutyric acid (GABA) production using Bifidobacterium adolescentis
Source: Microb Cell Fact. 2022 Mar 7;21:33. doi: 10.1186/s12934-021-01729-6 (PMC8903651; doi:10.1186/s12934-021-01729-6)
Supplement: Supplementary file 2 — Additional file 2: Table S1. GABA production and glutamate/GABA conversion ratio by different recombinants of Bifidobacterium. Table S2. Summary of GABA production ability by various microorganisms. Table S3. Independent variables and their coded and actual values were used in RSM optimization. Table S4. Three-level two-factor full factorial design arrangements and responses. Table S5. Analysis of variance (ANOVA) for regression analysis of B. adolescentis JCM 1275/pKKT427::Pori-gadBC. Table S6. Analysis of variance (ANOVA) for regression analysis of B. adolescentis JCM 1275/pKKT427::Pgap-gadBC. [file 12934_2021_1729_MOESM2_ESM.docx]

Supplementary Table 1. GABA production and glutamate/ GABA conversion ratio by different recombinants of *Bifidobacterium*

| Bacterial name | GABA (mM) | Conversion ratio (%) |
| --- | --- | --- |
| *B. adolescentis* 4-2 | 14 ± 1 | 21 ± 0.8 |
| *B. adolescentis* JCM 1275/pKKT427::P*_ori_*-*gadBC* | 70 ± 4 | 103 ± 9 |
| *B. adolescentis* JCM 1275/pKKT427::P*_gap_*-*gadBC* | 72 ± 2 | 105 ± 3 |
| *B. adolescentis* JCM 1275/pKKT427::P*_BLt43_*-*gadBC* | 71 ± 2 | 103 ± 4 |
| *B. longum* 105A/pKKT427::P*_ori_*-*gadBC* | 4 ± 1 | 6 ± 1 |
| *B. longum* 105A/pKKT427::P*_gap_*-*gadBC* | 66 ± 2 | 97 ± 3 |
| *B. longum* 105A/pKKT427::P*_BLt43_*-*gadBC* | 57 ± 3 | 84 ± 5 |
| *B. infantis* JCM 1222 /pKKT427::P*_ori_*-*gadBC* | 1 ± 0.1 | 1 ± 0.2 |
| *B. infantis* JCM 1222 /pKKT427::P*_gap_*-*gadBC* | 64 ± 3 | 94 ± 4 |
| *B. infantis* JCM 1222 /pKKT427::P*_BLt43_*-*gadBC* | 62 ± 6 | 91 ± 9 |
| *B. minimum* JCM 5821/pKKT427::P*_ori_*-*gadBC* | 0.1 ± 0.1 | 0.2 ± 0.1 |
| *B. minimum* JCM 5821/pKKT427::P*_gap_*-*gadBC* | 64 ± 5 | 94 ± 7 |
| *B. minimum* JCM 5821/pKKT427::P*_BLt43_*-*gadBC* | 58 ± 2 | 86 ± 4 |

Supplementary Table 2. Summary of GABA production ability by various microorganisms

| Microorganism | GABA (g/L) | References |
| --- | --- | --- |
| *B. adolescentis* JCM 1275/pKKT427::P*_ori_*-*gadBC* | 42 | This study |
| *Escherichia coli* BL21 (DE3)/GADZ11 | 103 | [44] |
| *Lactobacillus lactis* NCDO 2118 | 42 | [45] |
| *Escherichia coli* W3110 | 12.4 | [46] |
| *Lactobacillus brevis* NCL912 | 103.7 | [47] |
| *Lactobacillus brevis* TCCC 13007 (resting cells) | 201.8 | [48] |
| *Lactobacillus brevis* BH2 | 20 | [49] |
| *Lactobacillus brevis* TCCC13007 | 61 | [50] |
| *Lactobacillus plantarum* CGMCC 1.2437T | 74.3 | [51] |
| *Lactobacillus rhamnosus* YS9 | 19.28 | [52] |
| *Lactococcus lactis* subsp. *lactis* B | 6.41 | [53] |
| *Corynebacterium glutamicum* | 77.6 | [54] |
| *Escherichia coli* BW25113 | 614 | [55] |
| *Streptococcus thermophilus* fmb5 | 9.7 | [56] |
| *Bacillus subtilis* | 5.2 | [57] |
| *Streptococcus thermophilus* QYW-LYS1 | 2.9 | [58] |

Supplementary Table 3. Independent variables and their coded and actual values used in response surface methodology optimization

| Independent variable | Symbol | Code-level of variables | | |
| --- | --- | --- | --- | --- |
|  |  | -1 | 0 | 1 |
| Initial pH | *X_i_* | 4.4 | 5.2 | 6.0 |
| MSG addition (mM) (%MSG-V/ MRS-V) | *X_ii_* | 135 (2%) | 270 (4%) | 408 (6%) |

‎

Supplementary Table ~~4~~. Three-level of two-factors full factorial design arrangements and responses.

| Trail No. | Factors | | GABA yield (mM) R1 | | GABA yield (mM) R2 | |
| --- | --- | --- | --- | --- | --- | --- |
|  | *Xi* | *Xii* | experimental | predicted | experimental | predicted |
| 1 | -1 | -1 | 108.895 | 117.03 | 115.723 | 107.86 |
| 2 | -1 | 0 | 166.627 | 182.90 | 128.887 | 141.44 |
| 3 | -1 | 1 | 219.237 | 219.23 | 101.113 | 99.82 |
| 4 | 0 | -1 | 117.748 | 113.67 | 122.088 | 127.88 |
| 5 | 0 | 0 | 142.180 | 154.72 | 178.025 | 188.30 |
| 6 | 0 | 1 | 159.987 | 166.26 | 175.610 | 173.51 |
| 7 | 1 | -1 | 128.048 | 125.98 | 135.830 | 136.79 |
| 8 | 1 | 0 | 130.767 | 142.23 | 202.949 | 224.04 |
| 9 | 1 | 1 | 133.284 | 128.96 | 233.616 | 236.10 |

(R1) *B. adolescentis* JCM 1275**/**pKKT427::P*_ori_*-*gadBC*

(R2) *B. adolescentis* JCM 1275**/**pKKT427::P*_gap_*-*gadBC*

Supplementary Table 5. Analysis of variance (ANOVA) for regression equation for *B. adolescentis* JCM 1275**/**pKKT427::P*_ori_*-*gadBC*

| Source | Sum of squares | df | Mean square | *F*-value | *P* *> F* |
| --- | --- | --- | --- | --- | --- |
| Model | 17259.4 | 5 | 3451.88 | 81.28 | 0.000 |
| *Xi* | 7729.8 | 1 | 5359.21 | 126.19 | 0.000 |
| *Xii* | 3513.1 | 1 | 123.54 | 2.91 | 0.114 |
| *Xi Xii* | 5182.7 | 1 | 5182.66 | 122.03 | 0.000 |
| *Xi^2^* | 588.2 | 1 | 588.18 | 13.85 | 0.003 |
| *Xii^2^* | 245.7 | 1 | 245.73 | 5.79 | 0.033 |
| Residual | 1343.6 | 12 | 42.47 |  |  |
| Cor.total | 17769.1 | 17 |  |  |  |

*Xi=*Initial PH, *Xii=*MSG concentration (mM); Cor. Total = corrected total; R^2^ = 97.13%, adjusted R^2^ = 95.94%, predicted R^2^= 93.69% .

Supplementary Table 6. Analysis of variance (ANOVA) for regression equation for *B. adolescentis* JCM 1275**/**pKKT427::P*_gap_*-*gadBC*

| Source | Sum of squares | df | Mean square | *F*-value | *P* *> F* |
| --- | --- | --- | --- | --- | --- |
| Model | 31590.6 | 5 | 6318.13 | 173.77 | 0.000 |
| *Xi* | 0.1 | 1 | 0.13 | 0.00 | 0.954 |
| *Xii* | 83.9 | 1 | 83.86 | 2.31 | 0.155 |
| *Xi Xii* | 6064.30 | 1 | 6064.30 | 166.79 | 0.000 |
| *Xi^2^* | 3812.7 | 1 | 3812.68 | 104.86 | 0.000 |
| *Xii^2^* | 123.4 | 12 | 123.42 | 3.39 | 0.090 |
| Residual | 436.3 | 17 | 36.36 |  |  |
| Cor.total | 32026.9 | 17 |  |  |  |

Xi=Initial PH, Xii=MSG concentration (mM); Cor. Total = corrected total; R2 = 98.64%, adjusted R2 =98.07%, predicted R2= 97.09%,‎
